# Cadmium Exposure and Pancreatic Cancer in South Louisiana

**DOI:** 10.1155/2012/180186

**Published:** 2012-12-24

**Authors:** Brian G. Luckett, L. Joseph Su, Jennifer C. Rood, Elizabeth T. H. Fontham

**Affiliations:** ^1^Epidemiology Program, School of Public Health, Louisiana State University Health Sciences Center, 2020 Gravier Street, 3rd Floor, New Orleans, LA 70112, USA; ^2^Department of Global Community Health and Behavioral Sciences, School of Public Health and Tropical Medicine, Tulane University, 1440 Canal Street, Suite 2300, New Orleans, LA 70112, USA; ^3^Epidemiology and Genomics Research Program, Division of Cancer Control and Population Sciences, National Cancer Institute, 6130 Executive Boulevard, Room 5140, MSC 7393, Bethesda, MD 20892, USA; ^4^Clinical Chemistry Core, Pennington Biomedical Research Center, 6400 Perkins Road, Baton Rouge, LA 70808, USA

## Abstract

Cadmium has been hypothesized to be a pancreatic carcinogen. We test the hypothesis that cadmium exposure is a risk factor for pancreatic cancer with a population-based case-control study sampled from a population with persistently high rates of pancreatic cancer (south Louisiana). We tested potential dietary and nondietary sources of cadmium for their association with urinary cadmium concentrations which reflect long-term exposure to cadmium due to the accumulation of cadmium in the kidney cortex. Increasing urinary cadmium concentrations were significantly associated with an increasing risk of pancreatic cancer (2nd quartile OR = 3.34, 3rd = 5.58, 4th = 7.70; test for trend *P* ≤ 0.0001). Potential sources of cadmium exposure, as documented in the scientific literature, found to be statistically significantly associated with increased risk of pancreatic cancer included working as a plumber, pipefitter or welder (OR = 5.88) and high consumption levels of red meat (4th quartile OR = 6.18) and grains (4th quartile OR = 3.38). Current cigarette smoking, at least 80 pack years of smoking, occupational exposure to cadmium and paints, working in a shipyard, and high consumption of grains were found to be statistically significantly associated with increased concentrations of urinary cadmium. This study provides epidemiologic evidence that cadmium is a potential human pancreatic carcinogen.

## 1. Introduction

South Louisiana has experienced persistently high mortality rates of pancreatic cancer since at least the 1950s [1], and mapping of mortality from pancreatic cancer in the United States has found significantly elevated rates in southern Louisiana [2]. From 2000 to 2004, south-central Louisiana, the Acadiana region, had the highest incidence rate in the state with 15.9 cases per 100,000 residents [3].

Cadmium is a known human carcinogen [4] and has been hypothesized as a cause of human pancreatic cancer [5]. There is animal model evidence to suggest that it is a plausible pancreatic carcinogen [6]. Cadmium induces transdifferentiation of pancreatic cells [5] and increases synthesis of DNA in the pancreas, possibly through increased synthesis of metallothionein [7]. Metallothioneins are cysteine-rich, low-molecular-weight proteins that bind to xenobiotic heavy metals such as cadmium in the detoxification process [8].

Pancreatic cancer patients were found to have significantly higher serum cadmium levels than noncancer patients in Egypt [9]. However, serum concentration of cadmium may not reflect chronic exposure, typical of many environmental carcinogenic processes, since the half-life of cadmium in blood is only 2 to 3 months [10]. Urinary cadmium concentrations have been found to be a better indicator of long-term cadmium exposure than blood levels due to accumulation of cadmium in the kidney cortex [11, 12].

Higher urinary cadmium concentrations have been found to be associated with increased risk of certain cancers. Menke et al. [13] found that higher creatinine-adjusted urinary cadmium levels were associated with increased cancer mortality among men in the NHANES III Mortality Study. Nawrot et al. [14] found that higher urinary cadmium concentrations were associated with significantly increased incidence of all cancers and lung cancer specifically in a Belgian population-based cohort, while Arisawa et al. [15] found a similar effect among a Japanese cohort. Increased urinary cadmium levels have also been found to be associated with increased risk of breast cancer [16, 17] and bladder cancer [18]. Increased cadmium exposure has been shown to be associated with increased risk of renal cancers [19], endometrial cancer [20], and prostate cancer [21], but these studies did not use urinary cadmium excretion as a biomarker.

Cigarette smoke is a known source of cadmium due to the ability of the *Nicotiana* species to concentrate cadmium. Cadmium oxide released during the burning of a cigarette is highly bioavailable with smokers demonstrating 2 to 3 times the amount of cadmium in their kidneys as nonsmokers [22]. Cigarette smoking is an established risk factor for pancreatic cancer [23–25] with a recent meta-analysis finding about a 75% increase in risk [26]. Although the specific tobacco carcinogens that are linked to pancreatic cancer are not well established [27], cadmium is one possibility. Each cigarette contains 1 to 2 micrograms of cadmium [28].

Phosphate fertilizers and sewage sludge used as fertilizer contain cadmium [29]. Cadmium chloride is used in the production of some pesticides [30]. Continued applications of fertilizers and pesticides have raised cadmium concentrations in Swedish top soil by approximately 30 percent in the period from 1918 to 1980 [31]. Grain dust may contain residual cadmium from fertilizers or pesticides and may contain fungicides using cadmium carbonate [30]. However, cadmium from fertilizer and pesticide applications may not remain isolated. A study of metals in household dust conducted in southern Louisiana found higher mean cadmium concentrations in both indoor and outdoor dust samples in rural households than in urban households [32]. Cadmium from natural sources or from anthropogenic contamination may make its way into well water [33].

Cadmium acetate is used in the refining process to remove mercaptans from crude oil and gasoline [34]. Cadmium has been found in automotive lubricating oils and in diesel oil [35]. Fossil fuel combustion releases cadmium into the air [30].

Cadmium sulfide is used in the manufacture of certain paints to resist blackening by hydrogen sulfide [30]. In addition to the application of cadmium paints, removal of cadmium paints by scraping or blasting may also expose workers [36].

Other metals may contain some cadmium impurities that are released when the metal is smelted, cast, welded, or soldered. Cadmium alloys are used for soldering aluminum [30]. Welders and pipefitters may be occupationally exposed to cadmium through welding fumes [36]. Persons employed in ship yards may also be occupationally exposed [36].

Portland cement contains cadmium [37], and cadmium may also be used as an anticorrosive coating or present as an impurity in galvanized coatings on rebar used in reinforced concrete.

For nonsmokers, food is the primary source of cadmium exposure [22]. Approximately two-thirds of dietary cadmium intake is from plant sources with the remaining one-third coming from animal products [22]. Previous research has found an increased risk of pancreatic cancer among persons residing in south Louisiana consuming large quantities of rice, grains, and pork [23].

Rice and seafood are staples of the south Louisiana diet and may increase exposure to cadmium in this population. Cadmium bioaccumulates in grains and shellfish through uptake of naturally occurring cadmium and cadmium introduced to the environment through industrial emissions, fossil fuel combustion, pesticides, and chemical fertilizers [11]. Cadmium has been detected in high levels in rice in many parts of the world [38, 39]. Shimbo et al. [40] and Watanabe [41] found a correlation between cadmium intake and urinary cadmium concentrations among Japanese women with high consumption levels of rice.

High concentrations of cadmium in mollusks and crustaceans have been demonstrated [41–43]. Elevated cadmium levels have been found in oysters, shrimp, crabs, and crawfish [42, 44, 45] and in rice from south Louisiana (unpublished data).

We test the hypothesis that chronic cadmium exposure is positively associated with pancreatic cancer risk. To address this hypothesis, we regressed disease status on urinary cadmium levels and potential sources of cadmium exposure in a case-control study in south-central Louisiana. We also examined the association between potential sources of cadmium exposure and urinary cadmium concentration to address whether the risk factors we found for pancreatic cancer could be due to cadmium.

## 2. Materials and Methods

We limited eligibility for cases and controls to noninstitutionalized individuals older than 20 years of age and resident in one of eight south Louisiana parishes (counties) at the time of diagnosis for cases or ascertainment for controls. This human subjects research was reviewed and approved by the Louisiana State University Health Sciences Center: New Orleans, Institutional Review Board. We asked attending physicians for permission before contacting their patients, and every interviewee gave us informed consent.

In collaboration with the Louisiana Tumor Registry, we rapidly ascertained incident cases of pancreatic cancer diagnosed between March 1, 2001, and May 31, 2005, from south Louisiana hospitals. Although we ascertained most cases within one month of diagnosis, 60 percent were already deceased or too ill to participate by the time we attempted to contact them. Seventy-eight percent of cases that were able to participate did so. Since biologic specimens were necessary, no proxy interviews were conducted.

We randomly selected a population-based control group from driver's license and state-issued identification card files for subjects under 65 years of age and from Medicare rolls for subjects 65 years of age or older. Fifty-four percent of the eligible controls we identified participated. Control subjects were frequency matched on age, race (but not ethnicity), and gender in a 2 to 1 ratio to cases. We did not individually match controls to cases on parish of residence to avoid overmatching on environmental exposures; but all controls were residents of the same 8-parish catchment area from which we ascertained cases.

We administered a standardized and previously validated questionnaire to both cases and controls and asked them to provide a one-time blood and urine sample at the time of interview. The questionnaire included demographic data, residential and occupational histories, personal and family medical histories, tobacco and alcohol use, and other relevant information. Also included was a food frequency questionnaire developed at the National Cancer Institute (NCI) which we modified for use in this particular population by adding foods that are common to the south Louisiana diet [46]. The modified Block-NCI questionnaire was used because it employs both frequency and serving size data (ascertained with a visual guide) in the computation of average food consumption. We asked respondents to report their usual diet one year prior to interview to account for dietary changes associated with pancreatic cancer.

The spot urine samples were analyzed at the Pennington Biomedical Research Center on a Perkin Elmer atomic absorption spectrophotometer equipped with a graphite furnace. We measured creatinine in the urine samples using a Beckman-Coulter DXC 600 Pro. Cadmium concentrations are reported here as micrograms per gram of creatinine (*μ*g cadmium/g creatinine).

Cajun ethnicity has previously been demonstrated to be associated with an increased risk of pancreatic cancer in this population [23]. We defined “Cajun” for the purposes of this study as respondents who self-identified themselves as ethnically Cajun and reported that the French language was spoken in their home when they were children. This definition eliminated those persons who identify themselves as Cajun due to cultural affinity. We considered both African-Americans and Whites who met these criteria to be Cajun.

We adjusted for cigarette smoking in the regression models using two measures: pack years (included as a continuous variable) and whether a subject was a current smoker (defined as having smoked cigarettes within three years of interview). Thus, former smokers were defined as quitting at least three years prior to interview. We chose these variable definitions of current and former smoker to capture the expected effect of relatively recent tobacco exposure on urinary excretion of cadmium.

In addition to matching and smoking variables, our models of disease status also included variables for completion of high school, the typical number of alcoholic drinks consumed per day, and whether the subject had any first-degree relatives (parent, sibling, or child) who had been diagnosed with pancreatic cancer. To evaluate whether there is an association between potential cadmium exposures and urinary concentrations of cadmium, we regressed urinary cadmium concentrations on potential sources of cadmium separately with adjustment for matching parameters, pack years, current smoking status, and body weight and height.

We used unconditional logistic regression to calculate odds ratios and confidence intervals for disease risk adjusting for established risk factors for pancreatic cancer and frequency matching variables. We used linear regression to estimate the contribution of potential cadmium sources to urinary cadmium concentrations. We found that the distribution of urinary cadmium concentrations was skewed to the right, but we were able to normalize them using a natural log transformation. We present here the exponentials of the means and coefficients for the natural log transformed values in order to make them comparable to the untransformed values also presented. Our tests for trend were performed by regressing the outcome of interest on the categorical medians instead of categorical dummy variables and including all of the same covariates as previously. All statistical analyses were done using the SAS 9.1 software [47].

## 3. Results

Table 1 presents the distribution of characteristics of cases and controls included in the study. Cases and controls did not vary significantly by those factors on which the control series was frequency matched: age at interview, race, and gender. Since pancreatic cancer has very poor survival, cases were interviewed within weeks of diagnosis. Therefore, age at interview is practically the same as age at diagnosis.

Of the characteristics listed in Table 1, educational attainment and any first-degree relative with pancreatic cancer were significantly associated with disease status when controlling for matching variables only. Controls were significantly more likely to have completed their high school diploma or a general equivalency diploma (GED) than cases. While 77 percent of controls had a high school diploma or GED, only 62 percent of cases had one. Lack of a high school education more than doubled the risk of pancreatic cancer in this sample (OR = 2.12, 95% CI = 1.09–4.12, not shown in table). Only three percent of controls reported a first-degree relative with pancreatic cancer compared to 10 percent of cases (OR = 3.59, 95% CI = 1.09–11.85, not shown in table).

### 3.1. Potential Nondietary Sources of Cadmium

Table 2(a) presents odds ratios for disease status with 95 percent confidence intervals for potential sources of nondietary cadmium exposure. Levels of urinary cadmium were categorized into incremental quartiles of 0.5 micrograms of cadmium per gram creatinine with the top quartile capturing any values greater than 1.5 *μ*g/g creatinine. The odds ratios indicate a clear monotonic increase in risk of pancreatic cancer with increasing urinary cadmium concentrations (test for trend  *P* ≤ 0.0001, not shown in table). Using the group with the lowest concentrations as the referent, the next concentration level group had an odds ratio of 3.34 (95% CI: 1.38–8.07), followed by an odds ratio of 5.58 (95% CI: 2.03–15.34) for the third quartile and 7.70 (95% CI: 3.06–19.34) for the highest levels of urinary cadmium concentration. Urinary cadmium data were not available for all subjects either because the subject did not provide a sample (*n* = 16) or did not provide a sufficient quantity for analysis (*n* = 5). The urinary cadmium datum for one control subject was excluded from analysis after being identified as an outlier using the studentized residuals approach.

Current smokers showed an elevated risk of pancreatic cancer, but it was not statistically significant (OR = 1.52, 95% CI: 0.59–3.94). Being a former smoker (quitting at least three years prior to interview) was associated with a nonsignificant increased risk of pancreatic cancer in this sample. Pack years smoked were categorized into 40 pack year intervals with 80 or more pack years the highest category (OR = 2.82, 95% CI: 0.82–9.66). No category of pack years was found to confer a statistically significant risk for pancreatic cancer nor was the test for trend across categories statistically significant (*P* = 0.34). Consumption of alcoholic beverages was not associated with increased risk of pancreatic cancer either measured as current alcohol consumption per day or peak lifetime per day consumption and regardless of adjustment for smoking.

Body mass index and prior diagnosis of diabetes, stomach ulcer, or pancreatitis were not significantly associated with pancreatic cancer in this sample. Subjects reporting any first-degree relative that had been diagnosed with any cancer were found to be at increased, although nonsignificant, risk of pancreatic cancer (OR = 3.04, 95% CI: 0.88–10.50). Being Cajun was associated with a nonsignificant increase in risk for pancreatic cancer (OR = 1.52, 95% CI: 0.76–3.03).

A history of living in a residence in which the primary source of drinking water was from a private well showed an elevated risk for pancreatic cancer but was not significant. Having ever lived on any type of farm was not associated with pancreatic cancer, nor was a history of living on a rice farm or a cotton farm. However, a history of living on a sugar cane farm was found to be a significant risk factor for pancreatic cancer (OR = 3.17, 95%: 1.44–6.97).

A self-reported history of residing within one mile of a petroleum refinery elevated the risk of pancreatic cancer in this sample but was not statistically significant (OR = 1.48, 95% CI: 0.50–4.43).

Self-reported occupational exposures to pesticides (whether on a farm or in any setting), paints, heavy metals, and automotive and diesel exhaust were not associated with an increased risk of pancreatic cancer. Occupational exposure to grain dust, cadmium, aluminum, concrete dust, and petroleum products elevated the risk of pancreatic cancer but were not statistically significant. An occupational history of working as a welder, pipefitter, or plumber was associated with an increased risk of pancreatic cancer in this sample (OR = 5.88, 95% CI: 1.33–26.01). An occupational history of working in a shipyard was not associated with significantly elevated risk (OR = 3.13, 95% CI: 0.70–13.96), nor was a history of working in a refinery (OR = 1.87, 95% CI: 0.27–13.19).

The estimated contributions to urinary cadmium concentration associated with potential nondietary sources of cadmium exposure are presented in Table 3(a). Current cigarette smoking was associated with a significant elevation in urinary cadmium concentration compared to never smokers, while being a former smoker was not associated with increased urinary cadmium concentrations. Compared to lifetime nonsmokers, urinary cadmium concentration increased with increasing pack years with a significant test for trend across pack year categories (*P* < 0.0001). However, only the top category of pack years, at least 80, was statistically significantly associated with elevated urinary cadmium concentration.

Living in a residence in which the primary source of drinking water was from a private well was not significantly associated with an increase in urinary cadmium concentration, nor was ever having lived on any type of farm or ever having lived on a rice farm, cotton farm, or sugar cane farm. Occupational use of pesticides, whether on a farm or in some other setting, was not significantly associated with increased urinary cadmium concentrations. Exposure to grain dust was not associated with increased urinary cadmium concentrations.

Occupational exposures to cadmium and paints were each significantly associated with increases in urinary cadmium concentration as were exposures to aluminum and concrete dust (not shown). Self-reported occupational exposure to cadmium was estimated to elevate urinary cadmium concentration by 4.8 *μ*g cadmium per gram of creatinine (*P* < 0.05), while exposure to aluminum was estimated to elevate urinary cadmium concentrations by 0.8 *μ*g cadmium/g creatinine (*P* ≤ 0.01). Exposure to heavy metals such as lead, chromium, and nickel was not significantly associated with an increase in urinary cadmium concentration.

Occupational exposure to automotive or diesel exhaust was not significantly associated with increased urinary cadmium concentration. However, a history of ever having lived within one mile of a petroleum refinery did significantly predict urinary cadmium concentration with an estimated increase of  2.5 *μ*g cadmium/g creatinine.

An occupational history of ever working in a shipyard was significantly associated with an increase in urinary cadmium concentration, but a history of ever having worked in a refinery or as a pipefitter, welder, or plumber was not.

### 3.2. Potential Dietary Sources of Cadmium

Table 2(b) reports odds ratios by quartiles of weekly servings for various foods commonly consumed in south Louisiana and for which there is evidence that the food may be a source of dietary cadmium.

Crab and crawfish consumption was not individually associated with pancreatic cancer risk. There was a significantly elevated risk associated with the third highest category of shrimp consumption (OR = 2.69, 95% CI 1.24–5.84), but the test of trend was not statistically significant. Consumption of all shellfish combined (shrimp, crabs, and crawfish) was significantly associated with increased risk of pancreatic cancer among subjects consuming at least two servings of shellfish per day compared to those subjects consuming less than one-half serving per day (OR = 2.61, 95% CI: 1.02–6.69) although the test of trend was not significant (*P* = 0.09). Consumption of fish showed elevated risks for increased consumption but was not significantly associated with disease status nor was the test for trend statistically significant. Consumption of all seafood combined (crabs, crawfish, shrimp, oysters, and fish) showed an increasing risk with increasing consumption, but the test for trend was only marginally significant (*P* = 0.06).

Increasing levels of beef consumption were associated with increasing risk of pancreatic cancer although none of the odds ratios were significant and the test for trend was only marginally significant (*P* = 0.06). Increasing levels of pork consumption were associated with disease status (OR 2nd: 2.21, 95% CI: 0.94–5.21, OR 3rd: 2.14, 95% CI: 0.78–5.84 and OR 4th: 4.44, 95% CI: 1.66–11.89) with a significant test for trend (*P* ≤ 0.01). Increasing levels of consumption of red meat (beef and pork) were significantly associated with increased risk of pancreatic cancer (test for trend  *P* ≤ 0.01). Consumption of at least 12 servings per week of red meat was found to increase the risk of pancreatic cancer in this sample by more than five hundred percent compared to subjects eating less than four servings per week (OR 4th: 6.18, 95% CI 2.28–16.76). Consumption of chicken and turkey was not found to be associated with pancreatic cancer in this sample.

Rice consumption shows increasing risk by quartile (OR 2nd: 1.52, OR 3rd: 1.64, and OR 4th: 3.58) with a significant test for trend (*P* = 0.02), although only the highest category of consumption, at least 10 servings per week, was statistically significant. Wheat products showed increased risk of pancreatic cancer at higher consumption levels but none were statistically significant nor was the test for trend significant. All grain products combined show significantly increasing risk with levels of consumption (test for trend *P* value = 0.01), although only the odds ratio for the top category of consumption, at least 30 servings per week, was statistically significant (OR = 3.38, 95% CI: 1.10–10.36).

Table 3(b) presents the results from the predictive model of urinary cadmium concentrations. The top category of consumption of wheat products (at least 15 servings per week) predicted an increase of 1.7 *μ*g cadmium/g creatinine compared to subjects who consumed less than five servings of wheat products per week and was statistically significant at the 0.05 level as was the test for trend. All grains combined (wheat, rice, corn, rye, and oats) showed monotonically increasing estimates of contribution to urinary cadmium concentrations, but only the top category of at least 30 servings per week was statistically significant. The test for trend across categories of consumption of all grains was statistically significant.

Pork consumption was not significantly associated with increased urinary cadmium concentrations, although the top category (at least 8 servings per week) approached significance (*P* = 0.06). Consumption of red meat (beef and pork combined) was not significantly associated with urinary cadmium concentrations although the top category (at least 12 servings per week) approached significance (*P* = 0.07).

Consumption of rice, legumes, fruits and juices, or vegetables was not found to significantly contribute to urinary cadmium concentrations in this sample, nor was consumption of shellfish, fish, beef, or poultry (not shown).

## 4. Discussion

This study is the first to our knowledge to establish an increased risk of pancreatic cancer with increased urinary cadmium concentrations: the measure considered to be most indicative of long-term cadmium exposure. The monotonically increasing risk of disease with incremental quartiles of 0.5 micrograms of cadmium per gram creatinine is evidence of an etiologic link between cadmium exposure and pancreatic cancer. Given the history of excess incidence of pancreatic cancer among the population of south Louisiana, cadmium exposure should be considered as a potential environmental carcinogen driving these rates.

Our results confirmed some findings from an earlier study of pancreatic cancer done between 1979 and 1983 in south Louisiana [48] using hospital-based controls. That study found that consumption of at least 31 servings of pork per month increased risk by 44%, at least 30 servings of rice per month increased risk by 55%, and at least 60 servings of bread and cereals per month increased risk by 64%. Falk et al. [48] found a significantly elevated risk associated with Cajun ethnicity and moderate, and heavy cigarette smoking was nonsignificantly associated with pancreatic cancer risk. This earlier study also found a significant protective effect for the consumption of fruits and juices among persons consuming at least 64 servings per month that was not found here. It should be noted though that the earlier study employed a much larger sample size made possible through next-of-kin interviews.

Both studies of this population found that sugar cane farming was statistically significantly associated with increased risk of pancreatic cancer. No specific exposure that might explain this observed association that is related to sugar cane farming but not rice or other crops is known at this time. A significant association was found between residential proximity to an oil refinery and urinary concentrations of cadmium, but since proximity was self-reported recall bias cannot be ruled out for this result. We find it surprising that cigarette smoking was not found to be a significant risk factor for pancreatic cancer in this sample despite being a significant contributor to urinary cadmium concentrations. This may be related to the small sample size and limited statistical power.

This study had limited statistical power to detect weak associations; however, the elevated risk of pancreatic cancer associated with urinary concentration of cadmium found in this study provides support for the hypothesis that cadmium plays a role in its etiology. Further, this study provides some evidence that previous findings of increased risk associated with high consumption levels of red meat and grains may be related to their cadmium content. Pork may have higher cadmium content than beef due to being exclusively grain-fed, whereas cattle are generally grazed until six months before moving to feed lots to be finished with grain.

Increased synthesis of metallothionein may represent a mechanism through which cadmium acts in pancreatic carcinogenesis [7]. Ohshio et al. [49] found that positive staining for metallothionein in pancreatic carcinomas was more often associated with liver metastases, worse histological grade of the tumors, and shorter survival in a sample of 75 pancreatic duct cell carcinomas from Japan. Therefore, it is possible that the cases that were deceased at the time of contact or were too ill to participate had higher cadmium exposures than those found in participating cases. If so, that would bias our risk estimates associated with urinary cadmium concentrations and potential sources of cadmium exposure toward the null.

The lack of clear correlation between consumption levels of potential dietary sources of cadmium and urinary cadmium concentrations may be explained by previous findings that dietary cadmium intake and urinary excretion are poorly correlated at low levels of cadmium intake but improve at higher levels [50, 51]. Individual uptake of dietary cadmium may vary by other dietary characteristics such as fiber content as well as individual physiology such as blood iron stores [22, 51, 52].

The Cajun population and especially the older age cohort that we examined here consume large amounts of seafood, rice, and pork which could increase their exposure to cadmium. Additional research into sources of cadmium exposure and the carcinogenic effects of cadmium in the pancreas is warranted.

## Figures and Tables

**Table 1:** Demographic characteristics of the study population.

Factor	Level	Cases		Controls	
		*n*	%	*n*	%
Gender	Male	35	50.7	78	49.4
	Female	34	49.3	80	50.6

	<60	19	27.5	46	29.1
Age	60–69	17	24.6	41	26.0
	70+	33	47.8	71	44.9

	White	54	78.3	125	79.1
Race	African-American	15	21.7	29	18.4
	Other	0	0.0	4	2.5

Cajun	No	18	26.1	51	32.3
	Yes	51	73.9	107	67.7

Education	< High school	26	37.7	37	23.4
	High school +	43	62.3	121	76.6

Any first-degree relative with pancreatic cancer	No	62	89.9	153	96.8
	Yes	7	10.1	5	3.2

**Table:** (a)

Exposure	Level	Cases		Controls		OR^a^	95% CI
		*n*	%	*n*	%		
Urinary cadmium	<0.5 *μ*g/g creatinine	10	14.5	71	44.9	ref	
	0.5 to <1 *μ*g/g creatinine	16	23.2	33	20.9	3.34	(1.38, 8.07)
	1 to <1.5 *μ*g/g creatinine	13	18.8	18	11.4	5.58	(2.03, 15.34)
	1.5+ *μ*g/g creatinine	24	34.8	19	12	7.70	(3.06, 19.34)
	Missing/outlier	6	8.7	17	10.8	n/a	

Tobacco							
	Never	24	34.8	69	43.7	ref	
Cigarette smoking	Former	19	27.5	58	36.7	0.75	(0.32, 1.77)
	Current	26	37.7	31	19.6	1.52	(0.59, 3.94)
Pack years	0	24	34.8	69	43.7	ref	
	>0–<40	27	39.1	57	36.1	1.23	(0.60, 2.51)
	≥40–<80	11	15.9	26	16.5	0.80	(0.32, 2.03)
	≥80	7	10.1	6	3.8	2.82	(0.82, 9.66)

Residential							
Well water	No	45	65.2	123	77.9	ref	
	Yes	24	34.8	35	22.2	1.51	(0.77, 2.97)
Ever lived on any farm	No	24	34.8	73	46.2	ref	
	Yes	45	65.2	85	53.8	1.56	(0.81, 3.01)
Resided w/in 1 mile of refinery	No	63	91.3	148	93.7	ref	
	Yes	6	8.7	10	6.3	1.48	(0.50, 4.43)

Occupational							
Occupational any pesticides	No	63	91.3	135	85.4	ref	
	Yes	6	8.7	23	14.6	0.47	(0.17, 1.31)
Occupational paints	No	51	73.9	109	69	ref	
	Yes	18	26.1	49	31	0.73	(0.34, 1.60)
Occupational cadmium	No	68	98.6	156	98.7	ref	
	Yes	1	1.5	2	1.3	1.69	(0.14, 20.39)
Worked as pipefitter/plumber/welder	No	62	89.9	155	98.1	ref	
	Yes	7	10.1	3	1.9	5.88	(1.33, 26.01)
Worked in shipyard	No	65	94.2	154	97.5	ref	
	Yes	4	5.8	4	2.5	3.13	(0.70, 13.96)
Worked in refinery	No	67	97.1	155	98.1	ref	
	Yes	2	2.9	3	1.9	1.87	(0.27, 13.19)

**Table:** (b)

Food	Weekly servings	Cases		Controls		OR^a^	95% CI	Test for trend
		*n*	%	*n*	%			
All shellfish	<0.5	8	11.6	36	22.8	ref		
	≥0.5–<1	9	13	21	13.3	2.01	(0.63, 6.40)	
	≥1–<2	15	21.7	37	23.4	2.18	(0.78, 6.07)	
	≥2	37	53.6	64	40.5	2.61	(1.02, 6.69)	0.0898

All seafood	<0.5	4	5.8	16	10.1	ref		
	≥0.5–<1	5	7.3	23	14.6	1.10	(0.24, 5.13)	
	≥1–<3	25	36.2	61	38.6	2.15	(0.60, 7.71)	
	≥3	35	50.7	58	36.7	2.87	(0.79, 10.47)	0.0562

Beef	<1	12	17.4	34	21.5	ref		
	≥1–<3	21	30.4	56	35.4	1.03	(0.43, 2.48)	
	≥3–<6	19	24.5	46	29.1	1.21	(0.49, 2.98)	
	≥6	17	24.6	22	13.9	2.36	(0.86, 6.49)	0.0579

Pork	<2	10	14.5	52	32.9	ref		
	≥2–<5	26	37.7	56	35.4	2.21	(0.94, 5.21)	
	≥5–<8	13	18.8	29	18.4	2.14	(0.78, 5.84)	
	≥8	20	29	21	13.3	4.44	(1.66, 11.89)	0.0058

All red meat	<4	10	14.5	51	32.3	ref		
	≥4–<8	25	36.2	48	30.4	2.53	(1.05, 6.07)	
	≥8–<12	10	14.5	37	23.4	1.27	(0.45, 3.56)	
	≥12	24	34.8	22	13.9	6.18	(2.28, 16.76)	0.0009

Rice	<1	7	10.1	31	19.6	ref		
	≥1–<5	25	36.2	70	44.3	1.52	(0.57, 4.01)	
	≥5–<10	16	23.2	33	20.9	1.64	(0.55, 4.87)	
	≥10	21	30.4	24	15.2	3.58	(1.17, 10.91)	0.0203

Wheat	<5	15	21.7	47	29.8	ref		
	≥5–<10	21	30.4	54	34.2	1.42	(0.63, 3.20)	
	≥10–<15	16	23.2	22	13.9	2.43	(0.96, 6.14)	
	≥15	17	24.6	35	22.2	1.87	(0.78, 4.48)	0.1282

All grains	<10	6	8.7	24	15.2	ref		
	≥10–<20	20	29	63	39.9	1.48	(0.50, 4.42)	
	≥20–<30	20	29	38	24.1	2.50	(0.83, 7.57)	
	≥30	23	33.3	33	20.9	3.38	(1.10, 10.36)	0.0112

^a^Models include: age at interview, race, gender, pack years, current smoking, alcoholic drinks per day, family history of pancreatic cancer, and completing high school.

**Table:** (a)

Exposure	Weekly servings	Creatinine-adjusted urinary cadmium (*μ*g/g creatinine)		
		Mean	Coefficient^a^	*P* value
Tobacco use				
	Never	0.4764	ref	
Cigarette smoking	Former	0.5599	0.9923	0.9705
	Current	1.1647	2.1871	0.0013
Pack years	0	0.4764	ref	
	>0–<40	0.5892	1.0813	0.6806
	≥40–<80	1.1207	1.4802	0.1229
	≥80	1.2859	2.1231	0.0242

Residential				
Drank well water	No	0.6686	ref	
	Yes	0.5620	0.7603	0.1138
Ever lived on any farm	No	0.5901	ref	
	Yes	0.6769	1.1257	0.4449
Resided w/in 1 mile of refinery	No	0.6099	ref	
	Yes	1.3879	2.5056	0.0052

Occupational				
Any pesticides	No	0.6211	ref	
	Yes	0.7567	1.2395	0.3424
Cadmium	No	0.6291	ref	
	Yes	1.5530	4.7979	0.0117
Paints	No	0.6064	ref	
	Yes	0.7131	1.6692	0.0057
Worked in shipyard	No	0.6234	ref	
	Yes	1.1034	2.2175	0.0403
Worked in refinery	No	0.6315	ref	
	Yes	0.9328	1.7401	0.2584
Pipefitter, plumber or welder	No	0.6342	ref	
	Yes	0.7069	1.3642	0.3775

**Table:** (b)

Food	Weekly servings	Creatinine-adjusted urinary cadmium (*μ*g/g creatinine)			
		Mean	Coefficient^a^	*P* value	Test for trend
All shellfish	<0.5	0.5910	ref		
	≥0.5–<1	0.7460	1.2048	0.4957	
	≥1–<2	0.6335	1.1813	0.4751	
	≥2	0.6304	1.1845	0.4311	0.6251

All seafood	<0.5	0.8146	ref		
	≥0.5–<1	0.5582	0.7748	0.4658	
	≥1–<3	0.6317	0.9090	0.7416	
	≥3	0.6347	0.9514	0.8648	0.6586

Beef	<1	0.6552	ref		
	≥1–<3	0.5571	0.8778	0.5393	
	≥3–<6	0.6778	1.0781	0.7274	
	≥6	0.7094	1.2335	0.4129	0.2175

Pork	<2	0.5437	ref		
	≥2–<5	0.6688	1.1852	0.3826	
	≥5–<8	0.5010	0.8883	0.6092	
	≥8	0.9080	1.5445	0.0620	0.1490

All red meat	<4	0.5488	ref		
	≥4–<8	0.6807	1.1506	0.4871	
	≥8–<12	0.5678	0.9704	0.8926	
	≥12	0.7709	1.5221	0.0688	0.0867

Rice	<1	0.5055	ref		
	≥1–<5	0.6323	1.1886	0.4283	
	≥5–<10	0.7591	1.1673	0.5356	
	≥10	0.6518	1.2893	0.3280	0.4723

Wheat	<5	0.5381	ref		
	≥5–<10	0.5402	1.0538	0.7833	
	≥10–<15	0.7042	1.1326	0.5897	
	≥15	0.9044	1.7108	0.0105	0.0066

All grains	<10	0.5516	ref		
	≥10–<20	0.5297	1.6985	0.6299	
	≥20–<30	0.6407	1.8978	0.2120	
	≥30	0.8960	2.4498	0.0089	0.0012

^a^Models include age at interview, race, gender, body weight, height, pack years, and current smoking status.
